# Scaling Dynamic Response and Destructive Metabolism in an Immunosurveillant Anti-Tumor System Modulated by Different External Periodic Interventions

**DOI:** 10.1371/journal.pone.0016115

**Published:** 2011-01-14

**Authors:** Yuanzhi Shao, Wenyong Hu, Weirong Zhong, Li Li

**Affiliations:** 1 School of Physics and Engineering, Department of Physics, Sun Yat-sen University, Guangzhou, China; 2 Department of Physics, Jinan University, Guangzhou, China; 3 State Key Laboratory of Oncology in Southern China, Sun Yat-sen University, Guangzhou, China; University of South Florida College of Medicine, United States of America

## Abstract

On the basis of two universal power-law scaling laws, i.e. the scaling dynamic hysteresis in physics and the allometric scaling metabolism in biosystem, we studied the dynamic response and the evolution of an immunosurveillant anti-tumor system subjected to a periodic external intervention, which is equivalent to the scheme of a radiotherapy or chemotherapy, within the framework of the growth dynamics of tumor. Under the modulation of either an abrupt or a gradual change external intervention, the population density of tumors exhibits a dynamic hysteresis to the intervention. The area of dynamic hysteresis loop characterizes a sort of dissipative-therapeutic relationship of the dynamic responding of treated tumors with the dose consumption of accumulated external intervention per cycle of therapy. Scaling the area of dynamic hysteresis loops against the intensity of an external intervention, we deduced a characteristic quantity which was defined as the theoretical therapeutic effectiveness of treated tumor and related with the destructive metabolism of tumor under treatment. The calculated dose-effectiveness profiles, namely the dose cumulant per cycle of intervention versus the therapeutic effectiveness, could be well scaled into a universal quadratic formula regardless of either an abrupt or a gradual change intervention involved. We present a new concept, i.e., the therapy-effect matrix and the dose cumulant matrix, to expound the new finding observed in the growth and regression dynamics of a modulated anti-tumor system.

## Introduction

Dynamic hysteresis is a kind of nonequilibrium phenomenon and abounds in nature, and it usually occurs in such dynamic systems as physics, chemistry, mechanics, biology, ecology and so on. For instance, the collaborative interacting spin system driven by an external field [1]–[4], a perturbed mechanical system [5] and the ecosystem modulated by environmental elements [6]–[9]. Generally, the dynamic response of a driven system lags behind the driving force due to the asynchronization between the response and driving force, which gives rise to a universal dynamic hysteresis and loop [1]. The area and shape of dynamic hysteresis loop depend upon the intrinsic factors of a dynamic system as well as the extrinsic counterparts simultaneously. The main intrinsic factors include, as for a collaborative interacting spin ensemble, the coupling intensity and symmetry of spin system; the extrinsic counterpart is concerned with versatile types of driving forces, e.g., a linear or nonlinear driving field, a gradual or an abrupt change field. Usually, the hysteresis loop area of a dynamic system driven by its dual field quantifies the energy dissipation of the dynamic system after a unit cycle of dual field [1], whereas the shape and symmetry of a loop reflect the symmetry-breaking and restoring of dynamic ordering in a dynamic system [1], [2], [10]. Both dynamic hysteresis and transition provide much insight into a nonequilibrium dynamic system, and relevant investigations have been conducted in plenty since 1990. Among those investigations, the nonequilibrium dynamic transition, especially the dynamic hysteresis scaling, becomes the major concern which those investigators focus on. Generally, the area *S* of a dynamic hysteresis loop relates to both the amplitude *h* and the frequency *f* of driving force in a universal power-law form [1], [3], i.e.,
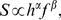
(1)where *α* and *β* are two scaling exponents, respectively.

Interestingly as well as importantly, in a biosystem without external driving there also exists ubiquitously a kind of spontaneous scaling law, i.e., the well-known allometric scaling law of metabolism, usually referred to as Kleiber's law for the situation of animals and plants, which relates the body mass *M* of a living being with its energy consumption *Y* in a power law as below:
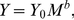
(2)where the *Y_0_* is a normalized prefactor relevant to a specific living creature, and the letter *b* is referred to a universal scaling exponent and actually served as an indicator of classification for different organisms [11]–[15]. The power-law scaling rule above reveals fundamentally the metabolic capability versus energy consumption emerging in a living-being in order to maintain its liveness. As the fundamental first principle in biology [13], the equation 2 holds true for both animal and plant species and discloses to some extent the similarity in their metabolism. For an organism, no matter what it belongs to, either animals or plants, the essential materials that the organism needs for sustaining its livingness are supplied and transported through space-filling fractal networks of branching tubes, which are of self-similarity and underlie the metabolic process of an organism [15]. The opinion of fractal networks mentioned above, however, was argued by Kurz et al [16]. According to the quantity of scaling exponent *b*, a variety of metabolic types have been classified as: for most organic species, the *b* takes an invariant value of *3/4*; the positive *1/4* of *b* conforms with the situations of times of blood circulation, embryonic growth and development, and life-span; the negative *1/4* of *b*, however, accounts for the rates of cellular metabolism, heartbeat and maximal population growth [15]. Another classification regarding the isometric and allometirc scaling types could be derived from whether *b = 1*or *b≠1*, respectively [14]. According to Dreyer [17], the origin of a power-law allometric scaling exponent has been ascribed to the existence of a supply-distribution system with a central source and a distribution of sources. A series of recent studies on allometric scaling, both theoretically and experimentally, present the arguments over the equation 2 and then revise it by involving more factors such as temperature, nonlinear term etc. to widely adapt to diverse biosystems in nature [18]–[25]. Readers may refer to the recent general review article for more information [24]. Actually, the scaling law of spontaneous metabolism in a living being has attracted the interdisciplinary concern, including biology, ecology, medicine, physics, mathematics, and so on.

The metabolism of a modulated biosystem, e.g., driven by a periodic external force, however, usually is adjusted to a certain rhythm and probably appears a new metabolic regime different from the spontaneous metabolism mentioned above. The variation of metabolism, due to the involvement of an external intervention, even results in some unfamiliar occurrences within a living being. Owing to the impact of war or disaster, a pregnant female undergoes a subtle change of metabolic level of incretion within her body, and she usually gives birth to a babygirl [25]. In the state of an emergency, enhancement of hormone level prompts the metabolic regulation in stress. It is of a fundamental significance to study the metabolic law of a living being, especially when it is modulated by an external intervention.

We simulated in this study the dynamic response of the metabolic growth and regression of tumors, which are modulated by an external periodic intervention similar to the scheme of a radiotherapy or chemotherapy. The networks of blood vessel, by analogy with the supply-distribution system of a central source as Dreyer described [17], act as a pipeline to carry the nutrient to sustain the growth of a tumor as well as the chemotherapeutant to kill a tumor, and it relates closely to the metabolism of a tumor under therapy. Based on the scaling metabolism without an external intervention described in most references, we focus on the external periodic intervention and the crucial role it plays on the metabolism of a tumor under therapy. Fractionated radiotherapy and chemotherapy are two major methods for the clinic treatment of cancer patients in advanced stage, and the dynamic regression and repopulation of cancer cells during treatment have a substantial impact on the curative effect, the prognosis and the recurrence and metastasis of cancer [26]. Intriguingly, does a simple and universal scaling formula exist to quantify the underlying metabolism between the regression of tumor cells and the dose consumption in chemotherapy or radiotherapy? The current paper will answer the question above on the base of our *in silico* research. We selected the dynamic differential equation (DDE) of tumor growth, which is of universality and well-proved experimentally by the enzyme dynamics [27]. We also introduced an external intervention term in original DDE to explore its new feature of dynamic evolution probably activated by an external intervention.

## Results

**Figure 1:**
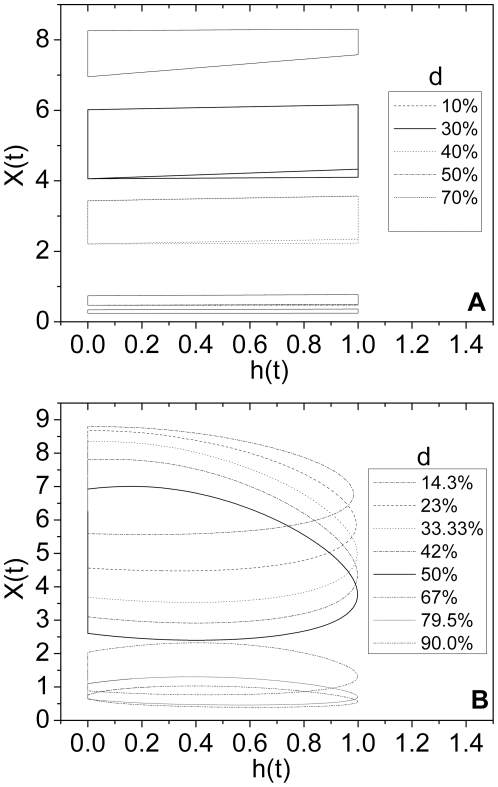
The dynamic hysteresis loops of the population density of treated tumors *x(t).* (A) an abrupt-change intervention (rectangular-wave) *h(t)*, (B) a gradual-change intervention (sinusoidal-wave) *h(t)*.

Figure 1 shows the dynamic hysteresis loops of the tumor population density *x(t)* against a rectangular wave *h(t)* (figure 1(a)) and sinusoidal wave *h(t)* (figure 1(b)) in a variety of duty-cycles of therapy, respectively. The dynamic hysteresis loop of a gradual-change sinusoidal intervention differentiates evidently its abrupt-change rectangular counterpart in shape and extend. The area of hysteresis loop *S* maximize at an intermediate duty-cycle of therapy *d*, referred to the *d_max_*, for two sorts of interventions above, suggesting that the intervention impacts most significantly on the tumor cells. The abrupt-change rectangular intervention attains its maximal area at a smaller *d_max_* value (∼30%) than its gradual-change sinusoidal counterpart (∼50%). Different external interventions do not alter the trend of the *S* versus the *d* other than the value of the *d_max_*. The maximal area of hysteresis loop signifies the realization of the most obvious destructive metabolic effect (apoptosis of treated tumor cells) along with the dose consumption of external intervention, viz. the most effective interplay between a medicament and the treated tumors. The dynamic hysteresis loop of the tumor population density against an intervention in figure 1 also indicates that the dynamic growth and regression of a tumor system, when modulated by an external intervention, is characterized by a sort of hysteretic metabolism.

**Figure 2:**
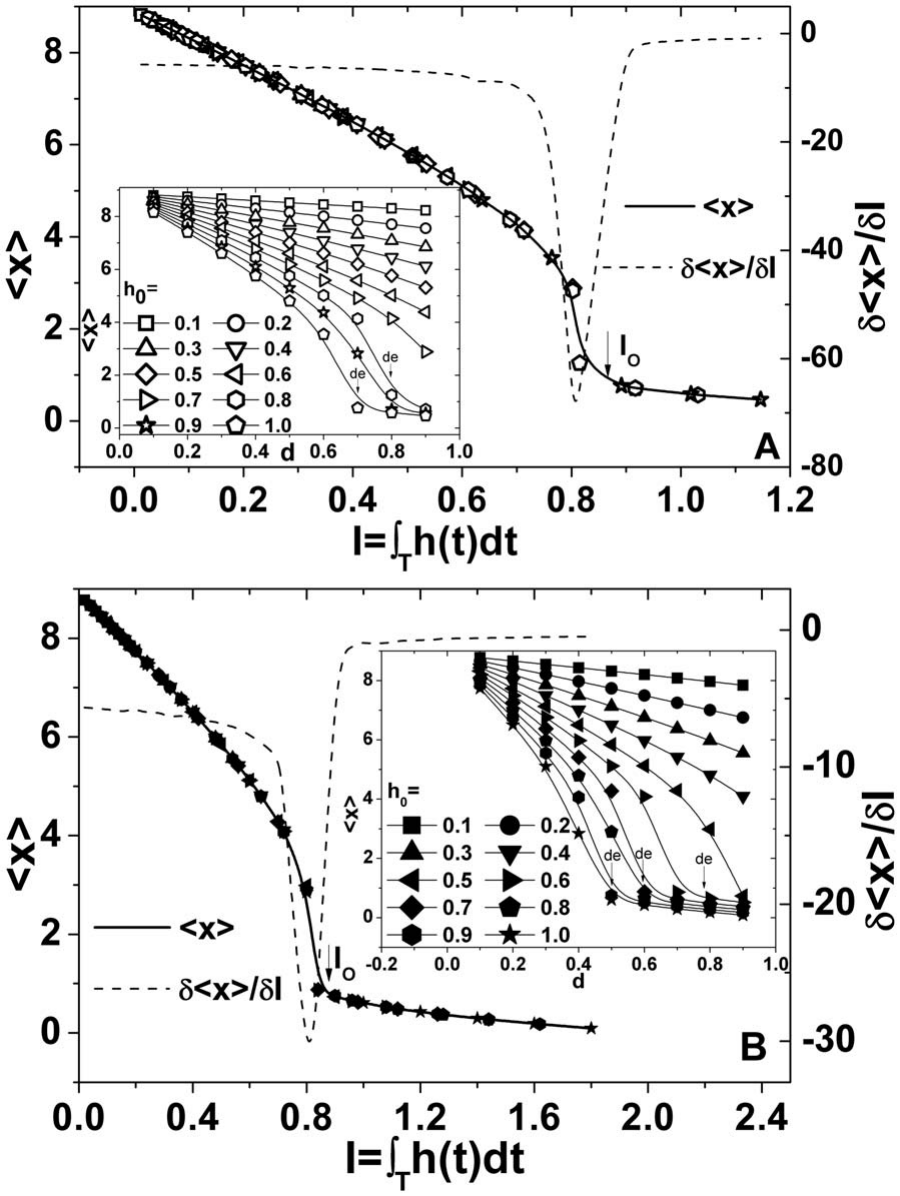
The dependence of average population density of treated tumors <*x*> on the dose cumulant of intervention *I* as well as the relevant first derivative *δ*<*x>/δI* per cycle. (A) sinusoidal and, (B) rectangular intervention in variety of the amplitudes of intervention *h_0_*. Inset: <*x*> versus the duty-cycle of therapy *d*.

Figure 2 depicts the dependence of average tumor population density <*x*> on the dose cumulant of intervention *I* per cycle, and the dose-effect relationship, i.e. *I* vs <*x*>, takes on a typical sigmoidal trend which is ubiquitous in biology and medicine. Larger dose cumulant *I* entails the decline of tumor cells, achieving a better treatment effect. Note that on the dose-effect curve there is a optimal dose cumulant *I_O_* (marked with a down-arrows) above which the average population density of treated tumors <*x*> maintains a quite low level and the dose cumulant affects the <*x*> slightly. Increasing the duty-cycle of therapy *d* leads to a distinctive decline of average population density <*x*>, but the declining trend, as demonstrated in the inset of figure 2, becomes obviously slight while the *d* is greater than a certain value to which we refer as the effective duty-cycle of therapy *d_e_* (marked with several down-arrows in inset). The effective duty-cycle of therapy *d_e_* is no longer observed in the case of small amplitude. Larger amplitude of intervention gains a smaller *d_e_*, indicating that an extended recovery period is necessary after a high-intensity treatment, which is in agreement with the common situation of clinic tumor therapy. It is also observed, according to the dot line in figure 2, that the largest derivative *δ<x>/δI* attains in the intermediate range of dose cumulant *I*, namely the tumor population density declines most drastically, an evidence that the destructive metabolic apoptosis of treated tumor cells occurs most significantly with a certain intermediate cumulant of dose consumption. Notice that the consistency that an intermediate duty-cycle of therapy *d* yields the largest area of dynamic hysteresis loop *S* as much as an intermediate dose cumulant *I* gives rise to the largest derivative *δ*<x>/*δI*.

**Figure 3:**
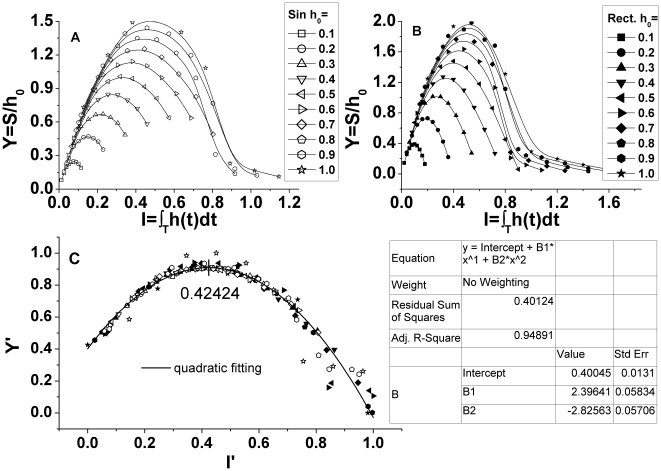
The destructive metabolic apoptosis of treated tumors *S/h_0_* versus different dose cumulants of intervention *I* per cycle. The amplitude of intervention *h_0_* ranges from 0.1 to 1.0. The open symbol (A) and solid symbol (B) were plotted singly for sinusoidal and rectangular intervention, respectively. The figure (C) represents the combined plot of rescaled data from the profiles of figure (A) and (B) by a quadratic conversion with a quadratic fitting curve, and the parameters in the table are fitting constants.

The dynamic hysteresis loop contains the general information of the variation of treated tumors and the modulation of interventional dose, and it characterizes a general destructive metabolic relation of the apoptosis of treated tumor cell *x(t)* with the dose consumption of medicament *h(t)* effecting on tumor cells within a period of treatment. Scaling the area of hysteresis loop *S* against the various amplitudes of external intervention *h_0_* from 0.1 to 1.0, we could work out the basic scaling trend of destructive metabolic apoptosis of treated tumors with a sort of intervention, and estimate relevant treatment capacity. The scaled area of hysteresis loop with amplitude *h_0_*, which we set *Y* = *S/h_0_*, signifies the variation, or more precisely a sort of destructive metabolism, of tumors subject to the intervention. Figure 3(a) and (b) show the profiles of the destructive metabolic apoptosis of treated tumors *Y* versus different kinds of dose cumulant of sinusoidal (a) and rectangular intervention (b) *I* per cycle, respectively, while the amplitudes of intervention *h_0_* range from 0.1 to 1.0. Rescaling the data of figure 3 (a) and (b) with the quadratic conversion of equation 3.1 and then reducing the rescaled data with equation 3.2∼3.3, we could further figure out the reduced form of the rescaled data *Y’* vs. *I’* and the figure 3(c) displays the reduced data of both sinusoidal and rectangular intervention as well as the quadratic fitting curve of the reduced data. The trend of rescaled data *Y’* vs. *I’* of two sorts of interventions, regardless of either a gradual or abrupt change, resembles each other considerably and reaches its maximum at an intermediate reduced dose consumption cumulant about *I’* = 0.4. The rescaled data could be well fitted in a quadratic formula by least square approximation as below:
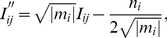
(3.1)for a quadratic formula *m x^2^+n x* with
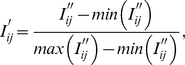
(3.2)

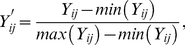
(3.3)where the symbol *i* denotes different values that the amplitude of intervention *h_0_* takes from 0.1 to 1.0, and the symbol *j* signifies various values that the duty-cycle of therapy *d* takes from 0.1 to 0.9; the combination of *i* and *j* is a matrix (9 columns, 10 rows currently) which we refer to as the dose cumulant matrix *I_ij_* since it contains both the intensity *h_0_* and the duration *d* of dose. The *Y_ij_* is referred to as the therapy-effect matrix and it functionally depends on the dose cumulant matrix *I_ij_*, expressed as *Y_ij_* = *F* (*I_ij_*) in brief.

## Discussions

In comparison with those preceding studies, the finding in current work is the new feature of a modulated anti-tumor system on the dynamic scaling hysteresis of dose-effectiveness: firstly, the most evident therapeutic effect *Y* occurs at an intermediate dose cumulant *I*; secondly, the dynamic scaling relation of dose-effectiveness complies with a quadratic-formula instead of the usual power-law form.

For the sake of simplicity physically and biologically, we assumed that merely the dose consumption of the dose accumulation *I* contributes to the destructive metabolic apoptosis of treated tumor cell, a simplicial causality of dose-effectiveness which underlies the study. As per the assumption, the destructive metabolic apoptosis of treated tumor cell is regarded as equivalent to the therapeutic effect *Y*, and it is easy to account for the reason why the most evident therapeutic effect occurs at an intermediate dose cumulant *I*. When the duty-cycle of therapy (or dose cumulant) is small, the lower therapeutic intensity can not afford to destroy the tumor cells substantially. Contrarily, the low level of tumor density under a quite large duty-cycle of therapy, as shown in figure 1, can not provide sufficient tumor cells to be treated, leading to a poor therapeutic effectiveness as well.

The result in figure 3(c) connotes somewhat a new sort of universal dynamic characteristic of an immunosurveillant anti-tumor system subject to two sorts of periodic external interventions which imitate the scheme of either a radiotherapy or chemotherapy. In spite of quite distinctive periodic external interventions involved, an intriguing universal quadratic-formula scaling relation that correlates the therapeutic effectiveness *Y* with the dose cumulant *I* was observed in the modulated anti-tumor system when both of them are scaled and reduced. The current anti-tumor model differentiates those models we mentioned above in two aspects. Intrinsically, the anti-tumor system in nature, or more generally a bio-system, can not probably behave with exact duality as a physical system of thermodynamics does as presented in equation 1. Extrinsically, unlike the spontaneous metabolism of a static bio-system without introducing any modulations as presented in equation 2, the current anti-tumor system is modulated dynamically by an intervention. We attributed the dynamic quadratic-formula scaling relation of the anti-tumor system to two new features mentioned above.

### Conclusions

The immunosurveillant anti-tumor system, when modulated periodically by either an abrupt- or gradual-change external intervention simulating the scheme of a radiotherapy or chemotherapy, exhibits the hysteretic dynamic response to the external intervention. The area of dynamic hysteresis loop characterizes a sort of dissipative-therapeutic relationship of the dynamically responding tumors under treatment with the dose consumption of accumulated external intervention per cycle of therapy. Through scaling approach, we worked out the theoretical therapeutic effectiveness of treated tumor, and found out that the scaled dose-effectiveness, namely the dose cumulant per cycle of intervention versus the therapeutic effectiveness, obeys a universal quadratic rule regardless of either an abrupt or gradual change intervention involved. The most evident therapeutic effect occurs at an intermediate dose cumulant of therapy. We also presented a new concept, i.e., the therapy-effect matrix and the dose cumulant matrix, to expound the new finding observed in the growth and regression dynamics of a modulated anti-tumor system.

## Methods

**Figure 4:**
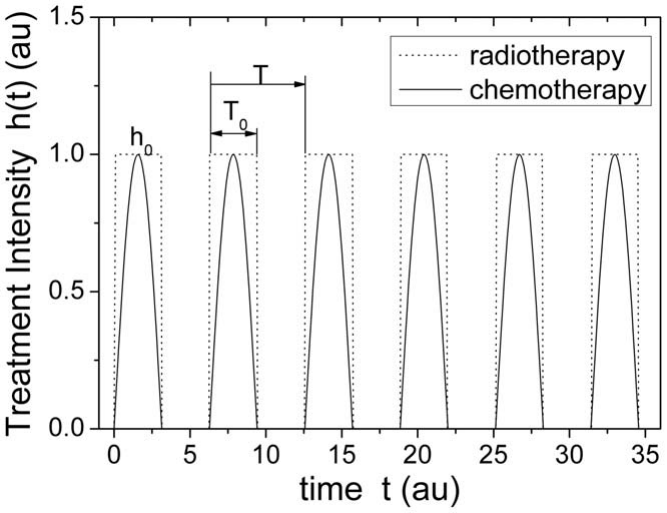
Schematic duty-cycle of therapy.

Concerning the growth and regression of tumor cells under immune surveillance against cancer, the dynamic transition between the normal and tumor cells can be modeled by the enzyme dynamic process and expressed schematically as below [27]:
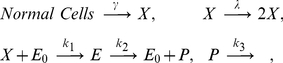
(4)in which the symbols *X, P, E_0_* and *E* symbolize cancer cells, dead cancer cells, immune cells and the compound of cancer cells and immune cells, respectively; the symbols *γ, λ, k_1_, k_2_* and *k_3_* are velocity coefficients. The above transition, which underlies our current study, indicates that normal cells may transform into cancer cells, and then the cancer cells reproduce, decline and die out ultimately. An equivalent single-variable deterministic dynamics differential equation, i.e., the well-established Logistic equation, can be derived from this model [28],
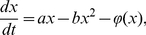
(5)where the *x(t)* and the *t* are the population density of tumor and the evolution time, respectively; the coefficients *a* and *b* stand for the linear per-capita growth rate of tumor cells and the carrying capacity of the environment, respectively. Term *ϕ(x)* quantifies the abilities of recognizability and attack which the immune cells act on the tumor cells, and its form is usually adopted as *βx^2^/(ε+x^2^)* [28]. Here, the parameter *β* and *ε* are the immune coefficient and the threshold at which the immune system initiates. A periodic external intervention *h(t)* which imitates a clinic therapeutic schedule was added in equation 5 and the final dynamic differential equation was rewritten as follows:
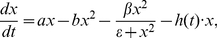
(6)where the periodic external intervention *h(t)* takes the form of either an abrupt change (rectangular wave) for radiotherapy or gradual change (sinusoidal wave) for chemotherapy, respectively, as illustrated in figure 4. The symbol *h_0_* denotes the amplitude of an external intervention, similar to the clinical therapy intensity of either chemotherapy or radiotherapy. The duration from 0 to *T* is defined as one cycle of treatment, and two partial durations, 0∼*T_0_* and *T_0_*∼*T*, are designated for the drug-taking and drug-suspending period, respectively. In the light of the ratio of drug-taking versus drug-suspending duration, we defined a new frequency-like characteristic parameter, *d = (T_0_/T)*×100%, as the duty-cycle of therapy. Actually, the parameter *h_0_* and *d* here are equivalent to *h* and *f*, respectively, in figure 2. The integral of the intervention *h(t)* over the duration *t*, *I* = ∫_T_ *h(t)δ t*, stands for the dose cumulant of sinusoidal (rectangular) wave within a cycle of treatment. We approached the equation 6 through numerical simulation, and worked out the solutions concerning the evolution of tumor population density *x(t)*, the time-average *x(t)* per cycle *<x(t)>*, and the extent of *x(t)* ranging from its maximum to minimum *Δx*.

Declaration of the value which these parameters take in our simulation: Throughout this paper we set *a = 1.0, b = 0.10, β = *1 and *ε = 1*. The therapy intensity *h_0_* and the duty-cycle of therapy *d* are given in relevant plots, respectively.

For the sake of simplicity, the treatment of tumors could be theoretically simplified as two basic processes: the apoptosis of tumor cells under treatment, regarded also as a kind of destructive metabolic process; the dose consumption of cumulative medicament, functioning as a kind of dissipation. Generally, medicament exerts its influence to tumor cells, and the dose consumption leads to the destructive metabolic apoptosis of treated tumors. Ideally, we assumed that the tumors under therapy are only treated by the external intervention (medicament), and therefore it is feasible for us to simply focus on the simplicial causality between the destructive metabolic apoptosis of treated tumors and the dose consumption. Because of the dynamic delay generally in nature, the response of tumor population density *x(t)* to an external intervention *h(t)* lags behind in phase, giving rise to a dynamic hysteresis loop of *x(t)* versus *h(t)* as shown in figure 1. The dynamic hysteresis loop of *x(t)* versus *h(t)*, however, could not be taken for the dissipated energy as usually in a thermodynamic system in that the tumor population density *x(t)* and the external intervention *h(t)* themselves can not relate to each other in duality essentially. As what the biochemical experiment demonstrates, the energy metabolism during the apoptosis of cell lines is complex and influenced by numerous factors [29]. Considering physically and biologically, we deem that the dynamic hysteresis above implies a sort of metabolic relation of the apoptosis of treated tumor cell *x(t)* with the consumption of medicament *h(t)* effecting on tumor cells within a period of treatment. The area of dynamic hysteresis loop *S* within a period *T* was figured out as below
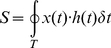
(7)

